# Effects of neighborhood built environment on cognitive function in older adults: a systematic review

**DOI:** 10.1186/s12877-024-04776-x

**Published:** 2024-02-27

**Authors:** Yiling Song, Yunxi Liu, Xiaotian Bai, Hongjun Yu

**Affiliations:** 1 Department of Physical Education, Tsinghua University, Beijing, 100084 China; 2 Graduate School of Commerce, Waseda University, Tokyo, 169-8050 Japan

**Keywords:** Neighborhood, Built environment, Cognitive function, Older adults, Systematic review

## Abstract

**Background:**

In the background of an aging population, the risk of cognitive impairment in the older population is prominent. Exposure to complex neighborhood built environments may be beneficial to the cognitive health of older adults, and the purpose of this study was to systematically review the scientific evidence on the effects of neighborhood built environments on cognitive function in older adults.

**Methods:**

Keywords and references were searched in Web of Science, Pubmed, PsycINFO, and MEDLINE. Studies examining the relationship between the built environment and cognitive function in older adults were included. The neighborhood built environment as an independent variable was classified according to seven aspects: density, design, diversity, destination accessibility, public transportation distance, blue/green space, and built environment quality. The cognitive function as the dependent variable was classified according to overall cognitive function, domain-specific cognitive function, and incidence of dementia. The quality of the included literature was assessed using the National Institutes of Health's Observational Cohort and Cross-Sectional Study Quality Assessment Tool.

**Results:**

A total of 56 studies were included that met the inclusion criteria, including 31 cross-sectional studies, 23 longitudinal studies, 1 cross-sectional study design combined with a case-control design, and 1 longitudinal study design combined with a case-control design. Most of the studies reviewed indicate that the built environment factors that were positively associated with cognitive function in older adults were population density, street connectivity, walkability, number of public transportation stops around the residence, land use mix, neighborhood resources, green space, and quality of the neighborhood built environment. Built environment factors that were negatively associated with cognitive function in older adults were street integration, distance from residence to main road. The relationship between residential density, destination accessibility, and blue space with cognitive function in older adults needs to be further explored.

**Conclusion:**

Preliminary evidence suggests an association between the neighborhood built environment and cognitive function in older adults. The causal relationship between the built environment and cognitive function can be further explored in the future using standardized and combined subjective and objective assessment methods, and longitudinal or quasi-experimental study designs. For public health interventions on the cognitive health of older adults, it is recommended that relevant authorities include the neighborhood built environment in their intervention programs.

**Supplementary Information:**

The online version contains supplementary material available at 10.1186/s12877-024-04776-x.

## Introduction

It is reported that about 8.5% of the world's population was aged 65 years and older in 2015, and this is expected to reach 16.7% by 2050 [1]. Population aging has become a global phenomenon and issue that is gradually becoming a topic of increasing concern for both developed and developing countries.

As the number of older adults increases, the risk of cognitive impairment increases. Cognitive impairment is characterized by reduced cognitive ability in one or more cognitive domains such as language, memory, executive function, computation and comprehension, and reasoning [2]. Dementia is the main cognitive impairment category, and one of the most common types of dementia is Alzheimer's disease (AD) [3]. Cognitive impairment not only affects the physical and mental health, daily living ability, and quality of life of older adults [4, 5], but also imposes a heavy economic burden on individuals, families, society, and healthcare [6]. Cognitive impairment in older adults has become an important public health issue. Therefore, to slow down cognitive decline and reduce the risk of dementia in older adults, the relevant authorities should actively propose appropriate intervention strategies.

Cognitive impairment can be influenced by a variety of modifiable risk factors, such as health behavior factors [7]. Many studies have shown significant correlations between physical activity, dietary behavior, sleep, smoking, alcohol consumption, and other lifestyle factors and the risk of cognitive decline and dementia [8–10]. In addition, research on the relationship between environmental factors and cognitive function in older adults has become an increasing focus of scholarly attention in recent years, particularly as the neighborhood built environment may affect cognitive function in older adults [11, 12]. Previous studies have pointed out that the built environment plays an important role in promoting healthy aging [13, 14]. For example, King et al. argued that the neighborhood built environment affects the physical activity levels of older adults, and the study showed that in less densely populated (and more suburbanized) neighborhoods, older adults would walk to errands less often [15]. In addition, Cassarino et al. suggest that exposure to complex, stimulating neighborhood environments may be one mechanism for delaying cognitive impairment and suggest that using the environment as a source of cognitive stimulation has the potential to contribute to a better understanding of successful aging as well as aging in place [16].

Built environment mainly refers to human-made buildings and spaces used for daily living, working, and recreating [17]. Measures of the built environment typically include density, design, diversity, destination accessibility, and proximity to public transportation, in addition to green space, aesthetics, and safety as some of the characteristics of the built environment [18, 19]. In the study on the relationship between the built environment and older adults' cognition, the results of Finlay et al. suggested that living in communities with higher local park accessibility, recreational facility availability, and commercial density may have a positive impact on cognitive function in older adults [20]. Green spaces may play an important role in promoting the health of older adults, and a perception of pedestrian safety may moderate the impact of green spaces on the health of older adults. However, the study by Crous-Bou et al. showed no significant association observed between green space around the community and cognitive composite indicators [21]. Different indicators of the built environment may have different effects on cognitive function in older adults.

Because the environment is an integral part of the various conditions that humans face in life, it can have lasting effects, especially on a specific group of older adults. However, previous studies still have gaps. First, the analysis of the relationship between different built environment elements and cognitive function has not been comprehensive enough. For example, built environment elements such as density, design features, and diversity may have different correlations with cognitive function in older adults, and few studies in the past have comprehensively and systematically synthesized the relationship between different built environment elements and cognitive function in older adults. Second, although some previous studies have shown that the built environment affects cognitive function in older adults, others have concluded that there is no significant relationship between the built environment and cognitive function in older adults. It is clear that the results of previous studies are mixed, and whether the built environment affects cognitive function in older adults still needs to be further explored. In addition, although some studies have systematically reviewed the relationship between the built environment and the health of older adults, fewer systematic reviews are focusing on the built environment and cognitive function in older adults. For example, Barnett et al. reviewed the relationship between the built environment and physical activity in older adults and found that safe, walkable, and aesthetically pleasing neighborhoods, with access to general and specific destinations and services, had a positive impact on older adults' participation in physical activity [22]. Tuckett et al.'s study also showed that the community's built environment affects the health behaviors and health status of older adults [23]. To compensate for the limitations of previous studies, this study systematically reviewed studies on the built environment and cognitive function of older adults, which not only helped to reveal the relationship between the built environment and cognitive function but also helped to provide some theoretical insights for future research.

In addition, active prevention and early treatment for high-risk older populations have become key to improving cognitive function and preventing and delaying the onset and progression of related diseases in older adults [24, 25]. Therefore, the purpose of this study was to systematically review the relationship between different built environment elements and cognitive functions of older adults and to summarize the effects of neighborhood built environment on cognitive functions of older adults. It is expected to provide scientific references for the improvement of cognitive function and the development of environmental interventions for older adults.

## Methods

This systematic review followed the recommendations provided by the Preferred Reporting Items for Systematic Reviews and Meta-Analysis guidelines [26].

### Literature search

Four electronic literature databases, Pubmed, Web of Science, MEDLINE, and PsycINFO, were systematically searched for literature. The search strategy is based on a combination of subject terms and free words. It is determined after iterative pre-screening and supplemented by manual searches, tracing citations back to the literature when necessary. The search period is from the start date of the creation of each database to July 7, 2023. The specific search strategy for PubMed, for example, is shown in Table 1.

**Table 1:** Pubmed Search Policy

Serial No	Search contents
#1	built environment* OR urban environment OR environment design OR urban design OR neighborhood* OR pedestrian environment OR street OR physical environment OR walkability OR residential environment OR community environment
#2	cognitive OR cognition OR dementia or Alzheimer* or demented
#3	older adult* OR elderly OR seniors OR aged OR older people OR older persons OR older citizens OR late life
#4	#1 and #2 and #3

### Inclusion and exclusion criteria

Inclusion criteria for the studies were as follows: (1) study participants: community-dwelling older adults; because the age thresholds used to define older adults varied across studies (e.g., people aged 65 and older are usually defined as older adults in developed Western countries, and older adults in developing countries are usually defined as people aged 60 and older), we included studies in which the authors described their study population as older adults; (2) study design: observational studies (e.g., cross-sectional studies or longitudinal studies), experimental studies, and qualitative studies; (3) independent variables: built environment (e.g., density, diversity, destination accessibility); (4) outcome variables: cognitive function or dementia (e.g., cognitive impairment, cognitive decline, risk of dementia); (5) language: papers published in English; and (6) type of article: peer-reviewed publications.

Exclusion criteria for studies were as follows: (1) no focus on the built environment; (2) studies that did not include cognition or dementia as an outcome variable; (3) repeated publications with poor quality assessment; (4) case reports, review literature, conference papers, and dissertations; and (5) non-English language papers.

### Study selection

Two researchers screened the literature independently according to the inclusion and exclusion criteria. First, the papers were initially screened by reading the title and abstract. Second, the full text was downloaded after obtaining the eligible literature, and full-text screening was performed. Finally, the two researchers compared the independently screened literature. For the literature with inconsistent screening results, the decision to include or not was to be made by a joint discussion with the third researcher.

### Data extraction and synthesis

Relevant information for the included literature was extracted independently by two researchers using a standardized form. The information extracted was as follows: (1) basic information: first author, year of publication, region/country, and setting; (2) basic characteristics of participants: age, sample size, the proportion of females, dementia restriction; (3) statistical analysis: statistical models; (4) other: study design, the spatial unit of analysis, types of measures to assess the built environment, detailed measure of built environment, types of measures to assess the cognitive function, detailed measure of cognitive function, the estimated effect of built environment on cognitive function, and key findings. Because heterogeneity in exposure and outcome metrics hindered Meta-analysis, we provide a narrative summary of common themes and findings from the included studies.

### Study quality assessment

Each included study's quality was evaluated using the National Institutes of Health’s Quality Assessment Tool for Observational Cohort and Cross-Sectional Studies (33). Each included study was required to undergo a quality evaluation containing 14 questions. For each question, "yes" responses received a score of one, while "no" responses received a score of zero. By computing the results for each criterion, a study's overall quality score is determined. Higher evaluation scores indicate higher quality of the included literature. The strength of the scientific evidence was measured using study quality assessment, however, the inclusion of research was not taken into consideration [27, 28]. The specific criteria and results of the literature quality evaluation can be found in Supplement 1.

## Results

### Search results

**Fig. 1:**
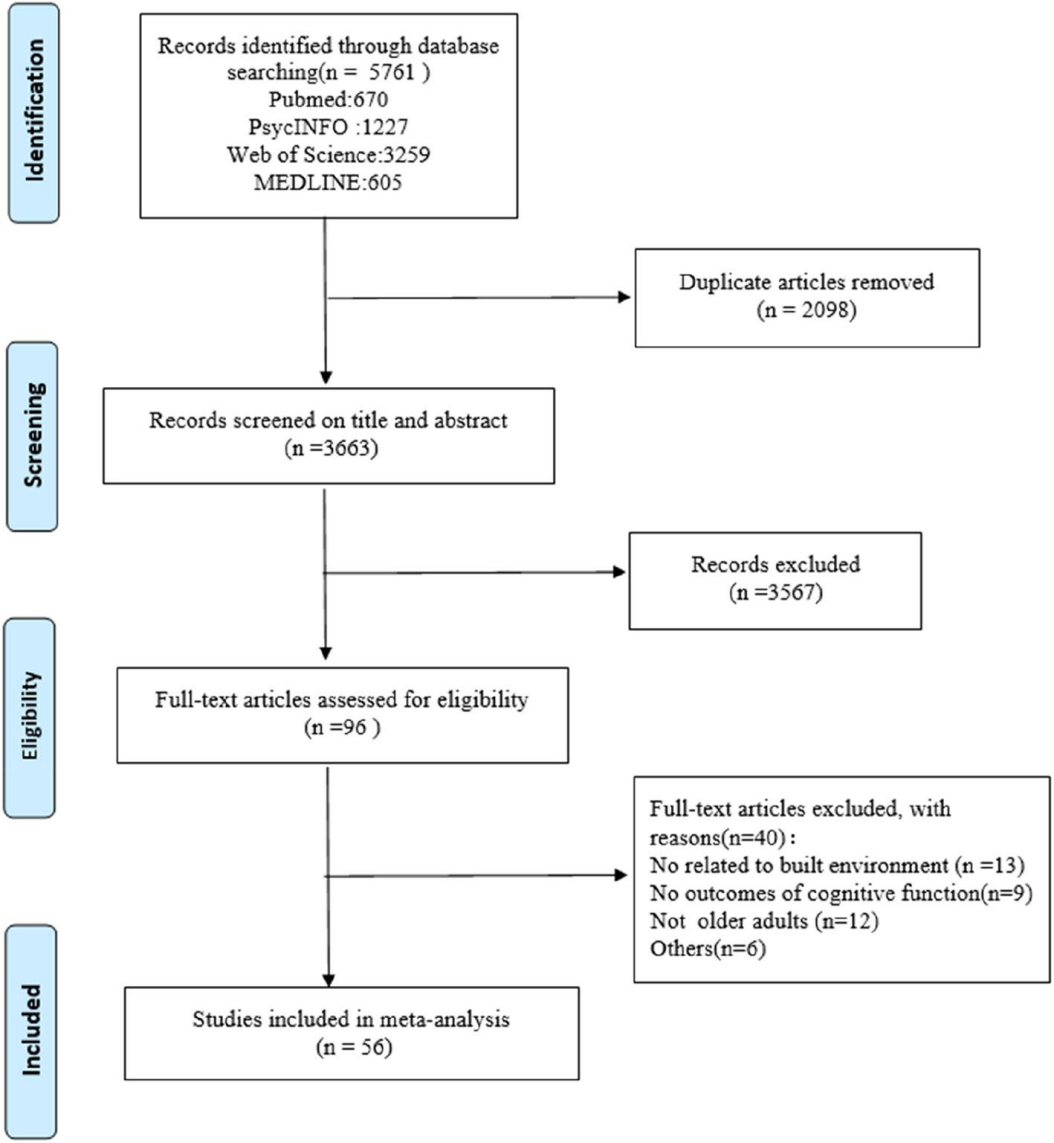
Flow diagram of study selection

A total of 5761 articles were retrieved from the database, and after removing duplicates, 3633 articles were obtained. By screening titles and abstracts, 3567 articles were excluded. After the full text of 96 articles was screened, 40 articles were excluded, and finally 56 articles were included in the study. Figure 1 shows the specific literature screening procedure.

### Basic characteristics of the included studies

A total of 56 studies were included in this study, as shown in Table 2, which summarizes the basic characteristics of the included studies and the quality assessment scores of the literature. The included studies were mainly published in 2012 and later, with more articles published in 2020 and 2021, with 13 and 11 articles, respectively; fewer articles were published between 2012 and 2017, with only 2 articles published in 2012 [29, 30], 3 articles published in 2015 [31–33], and 2 articles published in 2017 [34, 35]. The studies were mainly concentrated in the United States, China, the United Kingdom and Japan. There were three studies each from Hong Kong [36–38] and Taiwan [39–41], and only one study each from Singapore [42], Spain [21], and Ireland [43]. In terms of study design, 31 studies used a cross-sectional study design [21, 30, 32, 35–38, 41–64] and 23 studies used a longitudinal study design [20, 29, 31, 34, 39, 65–82]; in addition, one study used both a longitudinal study design and a case–control design [32], and one study used a cross-sectional study design and a case–control design [40].

**Table 2:** Basic characteristics of the included studies and the quality assessment of the studies

Study ID	First author (year)	Study location	Study design	Sample size	Age (years)	Female (%)	Dementia Restriction	Statistical model	Setting	Quality of the study (Score)
1	Clarke, 2012 [30]	USA	Cross-sectional	949	50 +	56.4	Not specified	Multilevel linear regression	Urban	7
2	Wellenius, 2012 [29]	USA	Longitudinal	765	65 +	63.9	MMSE > 18	GEE models	Not specified	8
3	Clarke, 2015 [31]	USA	Longitudinal	6,518	65 +	62	Not specified	Three-level linear model	Urban	8
4	Watts, 2015 [32]	USA	Longitudinal Case–control	64	74.41 ± 6.83	61.5	Early stage Alzheimer’s and healthy older adults	Multiple regression	Not specified	7
5	Wu, 2015 [33]	UK	Cross-sectional	2424	65 +	60.68	Not specified	Multilevel logistic regression	Urban	9
6	Chen,2017 [34]	Canada	Longitudinal	2165,268	55–85	53.2	Without dementia at baseline	Cox proportional hazard models	Urban and rural	8
7	Wu,2017 [35]	UK	Cross-sectional	7,505	65 +	54	Without dementia	Multilevel logistic regression	Urban and rural	8
8	Besser,2018 [44]	USA	Cross-sectional	4,539	45–84	53.2	Without dementia	Unadjusted and adjusted multivariate linear regression models	Urban	7
9	Brown,2018 [45]	USA	Cross-sectional	249,405	65 +	58.33	Not specified	Multilevel analysis	Not specified	7
10	Cherrie,2018	UK	Longitudinal	281	70–76	48	Not specified	"No effects" model and "saturated" model	Not specified	9
11	de Keijzer,2018 [66]	UK	Longitudinal	6,506	45–68	27.7	Not specified	Mixed effects models	Urban and rural	9
12	Lee, 2018 [46]	USA	Cross-sectional	2,260	57–85	52.04	Not specified	Ordinary least squares linear regression model	Urban and rural	7
13	Ng, 2018 [42]	Singapore	Cross-sectional	402	55–94	60.9	Not specified	Multi-variate regression with hierarchical models	Urban	8
14	Besser, 2019 [47]	USA	Cross-sectional	4,091	45–84	52.8	Without dementia	Linear regression models, multivariate models	Urban	7
15	Cherrie, 2019 [67]	UK	Longitudinal	281	70–76	48	Not specified	Multi-level linear regression models	Urban	8
16	Guo, 2019 [36]	Hong Kong	Cross-sectional	21,008	65 +	63.7	Not specified	Multilevel linear regression and multilevel logistic regression	Not specified	7
17	Koohsari, 2019 [48]	Japan	Cross-sectional	277	65–84	37.5	Not specified	Multivariable binary logistic regression and generalized linear models	Urban	7
18	Liu, 2019 [39]	Taiwan	Longitudinal	12,401	65 +	59.1	Not specified	Multilevel logistic regression	Urban and rural	8
19	Luo, 2019 [68]	China	Longitudinal	16,190	45 +	51.47	Not specified	Three-level linear growth curve model	Urban and rural	8
20	Tani, 2019 [49]	Japan	Cross-sectional	49,511	65 +	53.4	Without dementia	Cox proportional hazard models	Urban	8
21	Zaheed, 2019 [50]	USA	Cross-sectional	13,919	51 +	58.5	Not specified	Separate linear regression models	Not specified	7
22	Zhu, 2019 [69]	China	Longitudinal	38,327	65 +	58.91	Not specified	Linear regression, logistic regression, linear mixed effects regression, and mixed effects logistic regression	Urban and rural	8
23	Astell-Burt, 2020 [70]	Australia	Longitudinal	109,688	45 +	52.37	Without dementia	Multilevel discrete time-to-event history modelling	Urban	8
24	Besser, 2020 [51]	USA	Cross-sectional	4,084	45–84	53.6	Without dementia	Multilevel linear regression	Urban	7
25	Cassarino,2020 [43]	Ireland	Cross-sectional	224	65 +	72.7	Not specified	Bivariate analyses, negative binominal regression analyses	Not specified	7
26	Crous-Bou, 2020 [21]	Spain	Cross-sectional	958	45–74	63.9	Without dementia	General linear models	Urban	7
27	Estrella, 2020 [52]	USA	Cross-sectional	2,846	45–74	63.67	Not specified	Adjusted linear regression model	Urban	7
28	Finlay, 2020 [53]	USA	Cross-sectional	16,404	55–92	55	Not specified	Multilevel linear regression models	Urban	7
29	Katayama, 2020 [54]	Japan	Cross-sectional	3,786	65 +	51.5	Without dementia	Multinomial logistic regression	Suburban	7
30	Liu, 2020 [40]	Taiwan	Cross-sectional Case–control	26,206	65 +	54.7	Dementia and without dementia	Multilevel logistic regression	Urban and rural	7
31	Paul, 2020 [71]	Canada	Longitudinal	219,013	55–85	61	Without dementia at baseline	Mixed-effects Cox proportional hazards models	Urban	8
32	Sharifian, 2020 [72]	USA	Longitudinal	13,919	50 +	58.5	Not specified	Latent growth curve models	Not specified	8
33	Wu, 2020 [55]	UK	Cross-sectional	4,955	65 +	52.4	Not specified	Multilevel logistic regression	Urban and rural	7
34	Yuchi, 2020 [56]	Canada	Cross-sectional	647,447	45–84	52.65	Without dementia at baseline	Cox proportional hazard models, Conditional logistic regression	Urban	7
35	Zhu, 2020 [73]	China	Longitudinal	6,995	65 +	51.39	Not specified	GEE	Not specified	8
36	Aitken, 2021 [57]	USA	Cross-sectional	249,405	65 +	58.33	Not specified	Hierarchical regression models	Not specified	7
37	Bagheri, 2021 [58]	Australia	Cross-sectional	25,511	65 +	55.87	Without dementia	Multilevel mixed effect linear regression	Not specified	7
38	Besser, 2021 [74]	USA	Longitudinal	1,733	67.3 ± 8.3	53	Without dementia	Multivariable random intercept models	Urban	8
39	Besser, 2021a [75]	USA	Longitudinal	1,816	67 ± 8.2	52.7	Without dementia	Multivariable random intercept logistic models	Urban	8
40	Finlay, 2021 [20]	USA	Longitudinal	21,151	45 +	55.6	Not specified	Multilevel generalized	Urban and rural	8
41	Finlay, 2021a [76]	USA	Longitudinal	21,151	45 +	55.6	Not specified	Generalized additive multilevel models	Urban and rural	8
42	Hsu, 2021 [41]	Taiwan	Cross-sectional	1,356	65 +	50.1	Without AD	Multilevel mixed-effect model	Urban	7
43	Jin, 2021 [77]	China	Longitudinal	1,199	90 +	73.3	Not specified	Multivariable logistics regression models	Urban and rural	9
44	Motohiro, 2021 [78]	Japan	Longitudinal	485	66–75	55.1	Not specified	GEE	Rural	8
45	Tani, 2021 [79]	Japan	Longitudinal	76,053	65 +	53.4	Not specified	Multilevel survival models	Urban	8
46	Thierry, 2021 [59]	USA	Cross-sectional	80,236	65 +	56	Not specified	Multiple regression models	Urban and rural	7
47	Bai, 2022 [60]	China	Cross-sectional	745	60 +	65.6	Without dementia or AD	Multivariate linear regression	Urban	7
48	Cerin, 2022 [61]	Australia	Cross-sectional	4,141	61.1 ± 11.4	55.2	Not specified	Generalised additive mixed models	Urban	7
49	Finlay, 2022 [80]	USA	Longitudinal	21,151	67 ± 8.83	55.6	Not specified	Generalized additive multilevel models	Urban and rural	8
50	H. 2022	China	Cross-sectional	5,848	60 +	54.43	Not specified	Multilevel regression modelling	Urban and rural	8
51	Slawsky, 2022 [81]	USA	Longitudinal	3,047	75 +	53.7	Without dementia or AD	Cox proportional hazards models	Urban and rural	8
52	Sylvers, 2022 [63]	USA	Cross-sectional	10,289	73.4 ± 8.3	57	Not specified	Multilevel linear regression	Urban and rural	7
53	Chan, 2023 [37]	Hong Kong	Cross-sectional	1,873	65 +	53.5	Not specified	Multivariable linear regression model	Not specified	7
54	Godina, 2023 [82]	USA	Longitudinal	2,141	75.3 ± 4.8	56.8	Without dementia	Cox proportional hazard and logistic regression	Urban and rural	8
55	Ho, 2023 [38]	Hong Kong	Cross-sectional	2,077	65 +	55.45	Without dementia	Exposure-by-exposure regressions	Urban and rural	7
56	Kim, 2023 [64]	USA	Cross-sectional	814	65 +	57.7	Without dementia	Unadjusted and adjusted linear regression models	Urban and rural	7

*Notes*: *USA* United States of America, *UK* United Kingdom, *MMSE* Mini-Mental State Examination, *AD* Alzheimer’s disease, *GEE* Generalized estimating equation

The sample sizes of the included studies were generally large but varied considerably between studies. Twelve of the studies had a sample size of less than 1000, and the remaining studies had a sample size of 1000 or more. The studies were generally of older adults aged 65 years and older, and the proportion of women in each study ranged from 27.7% to 73.3%. There were also varying requirements for dementia restriction of study subjects, with several studies explicitly requiring that study subjects have no symptoms of dementia, but many studies did not explicitly require dementia symptoms in study subjects. In addition, a variety of statistical models were used in the included studies, including multilevel linear regression, generalized estimating equation, multilevel logistic regression, cox proportional hazard models, multilevel mixed effect linear regression, generalized additive multilevel models, unadjusted and adjusted linear regression models, and hierarchical regression models. Most studies were conducted in both urban and rural areas, and only one study was conducted in a rural area [78].

The literature quality assessment of the included studies showed that the mean score of the included studies was 7.57 (ranging from 7 to 9), with 28 articles (50%) scoring 7, 24 articles (42.86%) scoring 8, and 4 articles (7.14%) scoring 9. The detailed literature quality assessment results of the 56 studies are shown in Supplementary Table 1.

### Assessment measures of the built environment

Table 3 summarizes the measures of assessment of the built environment and cognitive function in the included studies. For measures of the built environment, the vast majority of studies used objective assessment measures (n = 4), 6 studies used subjective assessment measures [43, 50, 52, 59, 68, 72], and 4 studies used a combination of objective and subjective assessment measures [42, 49, 63, 64]. The objective assessment method of the built environment was mainly processed by using Geographic Information System (GIS) and satellite remote sensing images.

**Table 3:** Measures of built environment and cognitive function in the studies included in the review

Study ID	First author (year)	Spatial unit of analysis	Types of measures to assess the built environment	Detailed measure of built environment	Types of measures to assess the cognitive function	Detailed measure of cognitive function
1	Clarke,2012 [30]	AB = Census tracts	Objective measure:Census block group	Park area,Recreational centers, Institutional resources, Neighborhood disorders	Objective measure: Modified version of Telephone Instrument for Cognitive Status	Cognitive function
2	Wellenius, 2012 [29]	NA	Objective measure: ArcGIS software	Residential distance to major roadways	Objective measure: MMSE,HVLT-R,Recogntion, Clock-in-the Box,TMT	Cognitive function, Immediate recall, Delayed recall, Recognition,Language,Working memory, Attention and psychomotor speed, Set-shifting
3	Clarke,2015 [31]	AB = Census block group	Objective measure:Census block group	Presence of community centers, Access to public transit, Presence of cross walks, Presence of discontinuous sidewalk, Quality of public spaces	Objective measure: East Boston Memory Test,The symbol digit modalities test, MMSE	Immediate and delayed recall, Perceptual speed, General cognitive orientation and function
4	Watts, 2015 [32]	SPB = 0.5-mile buffer around participant's home address	Objective measure:ArcGIS software	Street connectivity, Street integration	Objective measure: MMSE, Wechsler Memory Scale, Free and Cued Selective Reminding Task, WAIS, Letternumber sequencing, DSC, Stroop Color Word Test	Cognitive function, Attention,Verbal memory
5	Wu, 2015 [33]	AB = UK postcode	Objective measure: UK Government Neighbourhood Statistics	Land use mix, Natural environment, Availability	Objective measure: MMSE	1.Cognitive impairment: MMSE < = 25; 2.Dementia
6	Chen, 2017 [34]	AB = 6-character postal-code addresses	Objective measure	Residential proximity to roads	Objective measure by diagnosis	Incidence of dementia
7	Wu, 2017 [35]	AB = UK postcode	Objective measure: UK Government Neighbourhood Statistics	Land use mix, Natural environment	Objective measure: MMSE	1.Cognitive impairment: MMSE < = 25; 2.Dementia
8	Besser, 2018 [44]	SPB = ¼-mile, ½-mile, and 1-mile buffer of residence	Objective measure	Social destination density, Walking destination density, Intersection density, Proportion land residential, Proportion land retail	Objective measure: CASI, DSF,DSB,DSC	Global cognition, Attention,Short term and working memory, Processing speed
9	Brown,2018 [45]	AB = Census block group	Objective measure:Advanced Spaceborne Thermal Emission and Reflection Radiometer satellite imagery	NDVI	Objective measure by diagnosis	Dementia risk
10	Cherrie, 2018 [65]	SPB = 1500m buffer around participants'home locations	Objective measure:ArcMap 10.1 GIS software	The percentage of parks within a 1500 m buffer zone surrounding residence	Objective measure: MHT No. 12	Cognitive aging in later life
11	de Keijzer, 2018 [66]	SPB = 500m buffer postcode centroids	Objective measure:the Moderate-Resolution Imaging Spectroradiometer onboard the Terra satellite	NDVI	Objective measure	Reasoning,Short-term memory, Verbal fluency
12	Lee, 2018 [46]	SPB = One block within home location	Objective measure	Neighborhood physical disorder index, Street disrepair index	Objective measure: MoCA-SA	Cognitive function
13	Ng, 2018 [42]	SPB = 500m buffer around the centroid of a neighborhood	1.Subjective measure: Neighborhood Environment Walkability Scale; 2.Objective measures: Neighborhood Environment for Active Transport, ArcGIS software	1.Subjective measure:residential density,street connectivity,land use mix-diversity,land use mix-access,infrastructure for walking or cycling,aesthetics,traffic safety,crime safety; 2.Objective measures:residential density,street connectivity,land use mix,walkability index,accessibility	Objective measure: RBANS	Global cognition, Immediate and Delayed Memory, Language, Attention and Visuospatial/Construction
14	Besser,2019 [47]	SPB = ½-mile radial area surrounding participant's home	Objective measure	Social destination density, Walking destination density, Intersection density, Proportion of land dedicated to retail	Objective measure: CASI,DSF,DSB,DSC	Global cognition, Working memory, Processing speed
15	Cherrie, 2019 [67]	SPB = 1500m and 500m buffer around participants'home and school; 300m and 100m buffer around the route from home to school	Objective measure:ArcGIS software	The area of public parks, Road traffic accident density	Objective measure: MHT No. 12	Cognitive performance
16	Guo, 2019 [36]	AB = Two levels of census tracts in Hong Kong	Objective measure: The Geo Community Database 2009 and the Geo Reference Database 2009	Neighborhood recreational environment, Neighborhood walkability, Neighborhood library accessibility	Objective measure: MMSE	1.Global cognitive function; 2.Dementia: MMSE < 24
17	Koohsari, 2019 [48]	SPB = 800m buffer area around participant's home	Objective measure:ArcGIS software	Street layout	Objective measure: MMSE	Cognitive function
18	Liu, 2019 [39]	NA	Objective measure:ArcGIS software	Parks,Greeneries,Square area, Playgrounds and sport venues, Community centers	Objective measure by diagnosis	Diagnosed AD
19	Luo, 2019 [68]	AB = Lowest administrative division in China	Subjective measure	Basic infrastructure, Outdoor spaces and buildings, Transportation,Availability of healthcare services	Objective measure: Telephone Interview of Cognitive Status battery	Cognitive function at baseline, Cognitive decline over time
20	Tani, 2019 [49]	SPB = 500m and 1,000m buffer around participant's residence	1.Objective measure: GIS software 2.Subjective measure: participant-reported	Availability of food stores	Objective measure by diagnosis	Dementia incidence
21	Zaheed, 2019 [50]	SPB = 20-min walk (about 1-mile) from participant's home	Subjective measure	Neighborhood physical disorder index	Objective measure: Immediate and Delayed Word Recall task, Animal fluency task	Episodic memory, Verbal Fluency
22	Zhu, 2019 [69]	SPB = 500m buffer area around participant's home	Objective measure: National Aeronautics and Space Administration's Terra satellite	NDVI	Objective measure: MMSE	Cognitive function
23	Astell-Burt, 2020 [70]	SPB = 1.6-km road network distance buffers of residence location	Objective measure	Total green space, Tree canopy, Open grass	Objective measure by diagnosis	Dementia incidence
24	Besser, 2020 [51]	SPB = 1-mile radial area surrounding participant's home	Objective measure: GIS software	Percentage park space in participant's neighborhood	Objective measure: CASI	Short-term memory, Long-term memory, Attention, Concentration, Orientation, Language, Verbal fluency, Visual construction, Abstraction/Judgment
25	Cassarino, 2020 [43]	SPB = Area around the house that could be covered by walking for 15 min	Subjective measure	Neighborhood pleasantness, Neighborhood streetscape	Subjective measure: The 25-item Cognitive Failure Questionnaire	Cognitive vulnerability: Cognitive failure, Sensory sensitivity
26	Crous-Bou, 2020 [21]	SPB = 300m buffer around participant's home	Objective measure: Map of Land Covers of Catalonia,2013	NDVI	Objective measure: WAIS-IV,Preclinical Alzheimer Cognitive Composite	Episodic memory, Executive function
27	Estrella, 2020 [52]	SJB = Self-perceived areas around where they live	Subjective measure: Self-reported perceived neighborhood environment	Neighborhood disorders	Objective measure: Brief Spanish–English Verbal Learning Test,Word Fluency Test of the Multilingual Aphasia Examination,WAIS-Revised,Digit Symbol Substitution Subtest	Global cognition, Verbal learning, Memory, Verbal fluency, Processing speed
28	Finlay,2020 [53]	AB = Census tracts	Objective measure: The National Establishment Time-Series database	Kernel density of retail fast food restaurants	Objective measure: WLL,WLD, AFT,LFT,MoCA	Cognitive function
29	Katayama, 2020 [54]	SPB = Within 5-min to 30-min walk from home	Objective measure	The Walk Score was based on distance to 13 categories of amenities	Objective measure: MMSE	Cognitive function
30	Liu, 2020 [40]	NA	Objective measure:ArcGIS software	1.Parks, greeneries, and square area; 2.Playgrounds and sport venues; 3.Community centers	Objective measure by diagnosis	Diagnosed AD
31	Paul, 2020 [71]	SPB = 250m buffer around participant's home	Objective measure: Landsat 5 satellite imagery	NDVI	Objective measure by diagnosis	Incidence of dementia
32	Sharifian, 2020 [72]	SPB = 20-min walk (about 1-mile) from participant's home	Subjective measure	Neighborhood physical disorder index, Street disrepair index	Objective measure	Episodic memory at baseline, Verbal fluency at baseline, Episodic memory change over time, Verbal fluency change over time
33	Wu, 2020 [55]	SPB = Within 400m and 800m of participant's residence	Objective measure: Google Maps and Open Street Map	Lifestyle (cafés, libraries, movie theatres, parks), Daily life (post offices, convenience stores), Healthcare (hospitals, pharmacies), Percentages of local green and blue spaces	Objective measure by diagnosis	Odds of dementia
34	Yuchi,2020 [56]	AB = Postcode	Objective measure: The CanMap road network, Landsat Enhanced Thematic Mapper Plus	Proximity to roads, NDVI	Objective measure by diagnosis	Incidence of non-Alzheimer's dementia, Incidence of Alzheimer's disease
35	Zhu, 2020 [73]	SPB = 500m buffer area around participant's home	Objective measure: National Aeronautics and Space Administration’s Terra Satellite	NDVI	Objective measure: MMSE	Cognitive impairment: MMSE < 24
36	Aitken,2021 [57]	NA	Objective measure:Advanced Spaceborne Thermal Emission and Reflection Radiometer satellite imagery	NDVI	Objective measure by diagnosis	Diagnosed AD
37	Bagheri, 2021 [58]	NA	Objective measure	Public open spaces, Walkability index, Length of major roads, Intersection density	Objective measure by diagnosis	Diagnosed dementia
38	Besser,2021 [74]	SPB = ½-mile and 1-mile buffer of residence	Objective measure	Neighborhood park space (park access),distance to nearest park	Objective measure: CASI,DSC	Global cognition,processing speed
39	Besser,2021a [75]	SPB = ½-mile and 1-mile buffer of residence	Objective measure	Social destination density, Walking destination density, Proportion of land dedicated to retail, Network ratio (street connectivity)	Objective measure: CASI,DSC	Global cognition, Processing speed
40	Finlay,2021 [20]	SPB = 1-mile radial area surrounding participant's home	Objective measure: NETS database	Civic and social organizations, Food and drinking placess, Senior centers	Objective measure: WLL, WLD, AFT,LFT,MoCA	Cognitive function
41	Finlay,2021a [76]	SPB = 1-mile radial area surrounding participant's home	Objective measure: NETS database	Parks,Fitness and sports recreation centers, Walkable destinations	Objective measure: WLL, WLD, AFT,LFT,MoCA	Cognitive function
42	Hsu, 2021 [41]	NA	Objective measure:City indicators from the governmental open data	Population density, Crime rat, Public library density, Green area for leisure purposes	Objective measure: MMSE	Cognitive function
43	Jin, 2021 [77]	SPB = 500m buffer area around participant's home	Objective measure: National Aeronautics and Space Administration's Terra Satellite	NDVI	Objective measure: MMSE	Cognitive function
44	Motohiro, 2021 [78]	SPB = 1000m buffer area around participant's home	Objective measure: GIS software	Residential density, Proximity to a community center	Objective measure:Cognitive Assessment for Dementia, iPad version 2	Cognitive function
45	Tani, 2021 [79]	NA	Objective measure:ArcGIS software	Sidewalk coverage	Objective measure by diagnosis	Incidence of dementia
46	Thierry, 2021 [59]	NA	Subjective measure	Cleanliness,Aafety,Social cohesion	Objective measure: Telephone Interview Cognitive Screen	Cognitive function
47	Bai, 2022 [60]	SPB = 800m buffer area around participant's home	Objective measure: QGIS software	Main roads, Secondary roads, Other roads	Objective measure: MMSE	Cgnitive function
48	Cerin, 2022 [61]	SPB = 1-km buffer area around participant's home	Objective measure: GIS software	Population density, Street intersection density, Land use mix, Percentage of parkland and blue spaces	Objective measure:California Verbal Learning Test, Symbol–Digit Modalities test	Cognitive function
49	Finlay, 2022 [80]	NA	Objective measure: NETS database	Arts sites, Civic and social organizations, Coffee shops and fast-food restaurants, Grocery stores, Higher education campuses, Libraries,Museums,Recreation centers, Religious organizations, Senior centers	Objective measure: WLL, WLD, AFT,LFT,MoCA	Cognitive function
50	H. 2022	SPB = 800m buffer area around participant’s home	Objective measure	The nearest facilities, Green/Blue spaces	Objective measure: MMSE	Cognitive function
51	Slawsky, 2022 [81]	SPB = 2-km buffer area around participant’s home	Objective measure	NDVI,Percent park overlap within a 2-km radius, Linear distance to nearest park	Objective measure by diagnosis	Diagnosed dementia
52	Sylvers, 2022 [63]	NA	Objectively measured and subjective perceptions	1.Objectively measured: Count Open Parks,Street Connectivity; 2.Subjective perceptions:problems with traffic and quality of parks	Objective measure:Consortium to Establish a Registry for Alzheimer’s disease,WLL, WLD,Animal Fluency Test	Cognitive function
53	Chan 2023 [37]	SPB = 200m buffer area of participant's home	Objective measure:ArcGIS software	Land-use mix, Public transport terminals, Community centers, NDVI,Commercial facilities, Cultural facilities, Active leisure facilities, Area of public open space, Accessibility of public open space	Objective measure:Cantonese Chinese MoCA	Cognitive function
54	Godina, 2023 [82]	SPB = 1-km and 5-km buffer area around participant's home	Objective measure:1992 National Land Cover Dataset	Greenspace	Objective measure by diagnosis	Diagnosed dementia
55	Ho, 2023 [38]	SPB = 200m and 500m buffer area of participant's home	Objective measure:2016 SPOT-6 multispectral satellite image, Hong Kong's 1:1000 topographic, Hong Kong's GeoCommunity Database 3.0	Greenery,Walkability,Accessibility	Objective measure by diagnosis	Diagnosed dementia
56	Kim, 2023 [64]	SPB = 800m buffer area of participant's home	1.Objective measure: GIS software; 2.Subjective measure:the Physical Activity Neighborhood Environment Scale	Walkability	Objective measure: CASI	Cognitive function

*Notes*: *MMSE* Mini-Mental State Examination, *AD* Alzheimer’s disease, *WAIS* Wechsler Adult Intelligence Scale, *GIS* Geographic Information System, *DSC* Digit Symbol Coding, *DSF* Digit Span Forward, *DSB* Digit Span Backward, *CASI* Cognitive Abilities Screening Instrument, *MHT* Moray House Test, *MoCA* Montreal Cognitive Assessment, *NDVI* Normalized Difference Vegetation Index, *NETS* National Establishment TimeSeries, *TMT* Trail Making Test, *HVLT-R* Hopkins Verbal Learning Test-Revised, *RBANS* Repeatable Battery for the Assessment of Neurocognitive Status, *AB* Administrative Boundary, *SPB* Spatial Boundary, *WLL* Word List Learning, *WLD* Word List Delayed Recall, *AFT* Animal Fluency Test, *LFT* Letter Fluency Test, *UK* United Kingdom, *NA* No Clear Definition

The objective built environment variables included in the study were grouped into six areas based on the elements of the built environment: (1) density—including population density, residential density; (2) neighborhood built environment design—including street connectivity, street integration, walkability index, neighborhood walkability, street layout, etc.; (3) public transportation distance—including the number of public transportation stops, distance from homes to major roads, distance from homes to roads, etc.; (4) destination accessibility—including accessibility of community libraries, accessibility of medical services, accessibility of food stores, accessibility of community centers, etc.; (5) neighborhood built environment diversity—including land use mix, recreation centers, community centers, basic medical facilities (hospitals, clinics, pharmacies, etc.), food and beverage venues, senior centers, fitness and sports recreation centers, coffee and fast food restaurants, grocery stores, higher education campuses, libraries, museums, recreation centers, commercial facilities, cultural facilities, etc.; (6) Blue and green spaces—including park areas, green spaces, tree canopies, open grassy areas, and blue waters, etc.; (7) Neighborhood built environment quality—including street disrepair index, cleanliness, neighborhood physical disorder, etc.

The subjective built environment was assessed mainly using questionnaires administered to the participants, such as the Neighborhood Environment Walkability Scale, self-reported perceived neighborhood environment, etc. The variables of subjective built environment include neighborhood physical disorder, Street disrepair index, Cleanliness, safety, neighborhood pleasantness, neighborhood streetscape, etc.

### Assessment measures of cognitive function

As shown in Table 3, For the assessment of cognitive function, most studies have used objective assessment measures such as Mini-Mental State Examination (MMSE), Digit Symbol Coding (DSC), Digit Span Forward (DSF), Digit Span Backward (DSB), Montreal Cognitive Assessment (MoCA), Wechsler Adult Intelligence Scale (WAIS), etc. Only one study measured cognitive function based on a self-report survey [43].

The variables of the outcome of cognitive function were divided into 3 categories, namely (1) overall cognitive function: cognitive changes over time, global cognition, cognitive impairment, cognitive performance, and Cognitive vulnerability; (2) domain-specific cognitive function: including immediate and delayed memory, recognition, language, working memory, attention and psychomotor speed, set-shifting, verbal memory, executive function, processing speed, reasoning, verbal fluency;(3) Incidence of dementia.

### Key findings

Table 4 summarizes the effects of a built environment on cognitive function in older adults. We summarize our key findings in the following seven areas.

**Table 4:** Estimated effects of built environment on cognitive function in the studies included in the review

Study ID	First author (year)	Estimated effects of built environment on cognitive function	Main findings of study
1	Clarke,2012 [30]	No effect was found for the proportion of recreation centers or park space in a person's neighborhood, but living in a neighborhood with a higher density of institutional resources was associated with higher cognitive functioning scores	Cognitive function: Institutional resources( +),Park area(0),Recreational centers(0), Neighborhood disorders (0)
2	Wellenius, 2012 [29]	Decreasing distance to major roadway was associated with statistically significantly poorer performance on the immediate and delayed recall components of the HVLT-R, TMT part B, TMT delta, and the letter and category fluency tests	Cognitive function: Residential distance to major roadways( +)
3	Clarke,2015 [31]	Residence in a block group with a community center and a public transit stop was associated with a slower rate of cognitive decline over time. Residents of neighborhoods in which public spaces were in poor condition had a faster rate of cognitive decline over time	Cognitive function: Community center( +), Public transit stop( +), Quality of public spaces( +),Presence of cross walks(0), Presence of discontinuous sidewalks(0)
4	Watts, 2015 [32]	1.Healthy controls:(1) Higher levels of neighborhood integration predicted greater declines in attention and verbal memory over the 2-year follow-up period. (2) Higher neighborhood connectivity predicted fewer declines in attention 2.Mild AD:Higher neighborhood integration predicted greater declines in attention over the 2-year follow-up	1.Healthy controls: (1) Neighborhood integration: Cognitive function(-),Attention(-), Verbal memory(-); (2) Neighborhood connectivity: Cognitive function( +),Attention( +),Verbal memory(0) 2.Mild AD: Neighborhood integration: Attention(-)
5	Wu, 2015 [33]	1.Living in areas with high land use mix was significantly associated with a nearly 60% reduced odds of dementia (OR: 0.4; 95% CI: 0.2, 0.8), but there was no linear trend for cognitive impairment 2.Increased odds of dementia (OR: 2.2, 95% CI: 1.2, 4.2) and cognitive impairment (OR: 1.4, 95% CI: 1.0, 2.0) were found in the highest quartile of natural environment availability	1.Cognitive impairment: Land use mix (0), Natural environment availability ( +) 2.Dementia:Land use mix (-), Natural environment availability ( +)
6	Chen, 2017 [34]	Living close to heavy traffic was associated with a higher incidence of dementia	Incidence of dementia: Residential proximity to roads( +)
7	Wu, 2017 [35]	1.Living in areas with high land use mix was associated with a nearly 30% decreased odds of cognitive impairment (OR¼0.72, 95% CI¼0.58, 0.89) 2.Living in areas with high natural environment availability was associated with 30% reduced odds of cognitive impairment (OR¼0.70, 95% CI¼0.50, 0.97)	1.Cognitive impairment: Land use mix (-),Natural environment availability (-) 2.Dementia: Land use mix (n),Natural environment availability (n)
8	Besser, 2018 [44]	Increasing social destination density, walking destination density, and intersection density were associated with worse overall cognition, whereas increasing proportion of land dedicated to retail was associated with better processing speed	1.Overall cognition: Social destination density(-),Walking destination density(-), Intersection density(-),Proportion land-residential(0); 2. Processing speed: proportion of land dedicated to retail( +),Proportion land-residential(0)
9	Brown,2018 [45]	There was a reduced risk of AD (by 18%) and depression (by 28%) for beneficiaries living in blocks that were 1 SD above the mean for greenness, as compared to blocks that were 1 SD below the mean	Dementia risk: NDVI(-)
10	Cherrie, 2018 [65]	Greater neighbourhood provision of public parks from childhood through to adulthood may help to slow down the rate of cognitive decline in later life	Cognitive function: Public parks availability ( +)
11	de Keijzer, 2018 [66]	An interquartile range increase in NDVI was associated with a difference in the global cognition z-score of 0.020 [95% CI: 0.003, 0.037; *p* = 0:02] in the 500-m buffer and of 0.021 (95% CI: 0.003, 0.039; *p* = 0:02) in the 1,000-m buffer over 10 y	Cognitive function: Residential surrounding greenness( +)
12	Lee, 2018 [46]	Individuals living in neighborhoods with poor built environment (street disrepair) have lower scores on the MoCA-SA than others (b = − 0.42; *p* < .05)	Cognitive function: Neighborhood physical disorder index(0), Street disrepair index(-)
13	Ng, 2018 [42]	The subjective measure of land use mix-diversity (standardized coefficient β = 0.161, *p* = 0.008) and GIS measure of walkability (β = 0.163, *p* = 0.002) were positively and significantly associated with RBANS global z-score, and immediate and delayed memory recall, visuospatial/ constructional ability and language	Global cognition: Residential density (0),Street connectivity (+ ,Objective measure),Walkability index ( +),Land use mix (0,Objective measure),Accessibility (0),Street connectivity (0,Subjective measure),Land use mix-diversity (+ ,Subjective measure), Infrastructure for walking and cycling (0),Aesthetics ( +),Traffic safety (0)
14	Besser,2019 [47]	Among APOE ε2 carriers, greater proportion of land dedicated to retail was associated with better global cognition, and greater SDD, WDD, intersection density, and proportion of land dedicated to retail was associated with better processing speed	1.Global cognition: Land dedicated to retail( +); 2.Processing speed: SDD( +), WDD( +), Intersection density( +),Land dedicated to retail( +)
15	Cherrie, 2019 [67]	Park availability during adolescence was associated with better cognitive aging at a concurrently low road traffic accident density (β = 0.98, 95% CI: 0.36 to 1.60)	Cognitive performance: Adolescent park availability( +), Childhood park availability(0),Traffic accident density (-)
16	Guo, 2019 [36]	Recreational environment was not a significant factor for older adults' cognitive function while library accessibility was. Neighborhood walkability was only related to dementia but not the cognitive function score	1.Cognitive function: Neighborhood recreational environment (0), Neighborhood walkability (0), Neighborhood library accessibility ( +); 2.Dementia: Neighborhood recreational environment (0), Neighborhood walkability (-), Neighborhood library accessibility (-)
17	Koohsari, 2019 [48]	There was a statistically significant negative association between street integration and the odds of having cognitive impairment	Cognitive function: Street integration( +)
18	Liu, 2019 [39]	Living in the areas with higher availability of playgrounds and sport venues was associated with a 3% decreased odds of AD (95% CI = 0.96–0.99), while higher density of elderly living alone was associated with a 5% increased odds of AD (95% CI = 1.01–1.11)	Cognitive function: The areas availability of playgrounds and sport venues( +), The density of elderly living alone(-)
19	Luo, 2019 [68]	Chinese older people who lived in neighborhoods with more handicap access, more bus lines, employment service had slower cognitive decline. Neighborhood basic infrastructures, number of days that roads were unpassable, outdoor exercise facilities, and average social activity participation were associated with baseline cognitive function in both rural and urban areas, but neighborhood environments had more impact on cognitive decline among rural older adults than urban older adults	1.Cognitive function at baseline: Basic infrastructure ( +), Days roads unpassable (-), Road tidiness (0), Handicapped access (0), Distance to bus stop (0), Number of accessible bus lines (0), Availability of healthcare services (0); 2.Cognitive decline over time: Basic infrastructure (+ , for urban only), Days roads unpassable (0), Road tidiness (0), Handicapped access (-, for rural only), Distance to bus stop (0), Number of accessible bus lines (-),Availability of healthcare services (0)
20	Tani, 2019 [49]	Compared with the highest quartile for objective availability of food stores, the hazard ratio adjusting for age and sex was 1.60 (95% CI = 1.43, 1.78) for the second-lowest quartile. Compared with the highest subjective availability of food stores, the hazard ratio was 1.74 (95% CI = 1.49, 2.04) for the lowest category	Dementia incidence: Availability of healthy food stores(-)
21	Zaheed, 2019 [50]	Perception of greater neighborhood physical disorder was significantly associated with worse episodic memory, while perception of lower neighborhood social cohesion was significantly associated with worse semantic fluency	Neighborhood physical disorder index: Episodic memory(-),Verbal Fluency(n)
22	Zhu, 2019 [69]	Each 0.1-unit increase in NDVI was associated with a 0.23-point increase in MMSE score (95% CI 0.16 to 0.29) in the linear regression, and an OR of 0.94 (95% CI 0.92 to 0.96) of having cognition impairment in the logistic regression. In the second analysis, looking at changes in NDVI and MMSE score, compared with the participants living in areas with an increase in NDVI, those living in areas with a decrease in greenness had an OR of 1.25 (95% CI 1.18 to 1.34) of a decrease in MMSE, and an OR of 0.90 (95% CI 0.84 to 0.96) of an increase in MMSE	NDVI: Cgnitive function( +), Cognitive impairment(-), Odds of cognitive decline(-)
23	Astell-Burt, 2020 [70]	1.Dementia risk was lower with more tree canopy (≥ 30% vs < 10% tree canopy incidence hazard ratio (IHR) = 0.86, 95%CI 0.75, 0.99) 2.Anti-dementia medication-based detection also indicated lower dementia risk with more open grass (≥ 20% vs < 5% IHR = 0.83, 95%CI 0.67, 1.03)	1.Anti-dementia medications based dementia incidence: Total green space ( +), Tree canopy ( +), Open grass (-) 2.Events based dementia incidence: Total green space (-), Tree canopy (-), Open grass (0)
24	Besser, 2020 [51]	Greater neighborhood park space 1-mile around the residence was associated with better processing speed in the overall sample (estimate: 0.48; 95% CI: 0.03, 0.92)	Processing speed: Neighborhood park space 1-mile around the residence( +)
25	Cassarino, 2020 [43]	Higher self-reported neighbourhood pleasantness was associated with lower cognitive vulnerability, particularly in older adults who lived in the most rural and urban areas (*p* = 0.006)	1.Cognitive failure: Neighborhood pleasantness (-, for living countryside or inner city only), Neighborhood streetscape (0) 2.Sensory sensitivity: Neighborhood pleasantness (0), Neighborhood streetscape (0)
26	Crous-Bou, 2020 [21]	No significant associations were observed between urban environmental exposures and the cognitive composite (*p* > 0.1)	Cognitive performance: Surrounding greenness(0)
27	Estrella, 2020 [52]	1.Women in the lowest quintile of perceived neighborhood problems had higher global cognition (β-0.48, 95% CI 0.03, 0.94, p trend 0.229) and memory scores (0.60, 95% CI 0.11, 1.09, p trend: 0.060) 2.Women in the highest quintile of perceived neighborhood social cohesion had lower global cognition (β—0.56, 95% CI—1.02,—0.09, p trend 0.004), verbal learning (B—1.01, 95% CI—2.00,—0.03, p trend 0.015), verbal fluency (B—2.00, 95% CI—3.83,—0.16, p trend 0.006), and processing speed (B—2.11, 95% CI—3.87,—0.36, p trend 0.009)	1.Global cognition: Neighborhood disorders (-, for women; 0, for men); 2.Memory: Neighborhood disorders (-, for women; 0, for men); 3.Neighborhood disorders: Verbal learning(0), Verbal fluency(0), Processing speed(0)
28	Finlay,2020 [53]	A positive association between kernel density of local eateries and cognitive functioning	Cognitive function: Kernel density of retail fast food restaurants( +)
29	Katayama, 2020 [54]	Neighborhood amenities were not significantly related to cognitive functioning	Cognitive function: Neighborhood amenities(0)
30	Liu, 2020 [40]	1.A significant reduction of 12% in the odds ratios of dementia in areas with higher availability of playgrounds and sport venues (OR 0.88, 95% CI 0.81–0.95) 2.Community center availability was also significantly associated with an 8% decreased odds for dementia (OR 0.92, 95% CI 0.87–0.99)	The odds ratios of dementia: The vailability of playgrounds and sport venues(-),Community center availability (-)
31	Paul, 2020 [71]	The hazard ratio per interquartile range increase in green space was 0.97 (95% CI: 0.96–0.98) for dementia	Incidence of dementia: NDVI(-)
32	Sharifian, 2020 [72]	Higher physical disorder was associated with worse initial episodic memory and verbal fluency through greater anxiety symptoms	Neighborhood physical disorder index: Episodic memory at baseline(-), Verbal fluency at baseline(-), Episodic memory change over time(0), Verbal fluency change over time(0)
33	Wu, 2020 [55]	Living far from daily life amenities was associated with higher odds of dementia in both CFAS II (1.47; 95% CI: 0.96, 2.24) and the 10/66 study (1.53; 95% CI: 1.15, 2.04), while living far from lifestyle (1.50; 95% CI: 1.13, 1.99) and healthcare amenities (1.32; 95% CI: 0.93, 1.87) was associated with higher odds of dementia only in the 10/66 study. A high availability of local green and blue spaces was not associated with dementia in either cohort yet living far from public parks was associated with lower odds of dementia in CFAS II (0.64; 95% CI: 0.41, 1.00)	Odds of dementia: Distance to café (+ , for those living in LMIC settings only), Distance to library (+ , for those living in LMIC settings only), Distance to movie theatre (+ , for those living in LMIC settings only), Distance to park (-, for those living in LMIC settings only), Distance to post office ( +), Distance to convenience store ( +), Distance to doctor/hospital (+ , for those living in LMIC settings only), Distance to pharmacy (0), Distance to recreational green (0), Distance to nature (0), Distance to blue space (0)
34	Yuchi,2020 [56]	Road proximity was associated with all outcomes (e.g. non-Alzheimer’s dementia hazard ratio: 1.14, [95% CI: 1.07–1.20], for living < 50 m from a major road or < 150 m from a highway)	Incidence of non-Alzheimer's dementia: Proximity to roads (-), NDVI (-) Incidence of Alzheimer's disease: Proximity to roads (0), NDVI (-)
35	Zhu, 2020 [73]	The protective effect of residential greenness (the highest vs. lowest quartile) on cognitive impairment was observed among the non-ε4 carriers (OR: 0.83, 95% CI: 0.72, 0.95), but not among the ε4 carriers (OR: 1.00, 95% CI: 0.74, 1.34)	Cognitive impairment: NDVI(-, for those non-ε4 carriers)
36	Aitken,2021 [57]	Compared to the lowest greenness tertile, the highest greenness tertile was associated with reduced odds of AD by 20% (odds ratio, 0.80; 95% CI, 0.75–0.85)	Greenness tertile: The odds of AD(-)
37	Bagheri, 2021 [58]	1-SD increases in public open space and socioeconomic status were associated with 3% (95% CI: 0.95, 0.98) and 1% decreases (95% CI: 0.98, 0.99) in dementia risk, respectively	Public open space: Dementia risk(-)
38	Besser,2021 [74]	Greater park access (equivalent to 10% more in ½-mile around home) was associated with maintained/improved CASI score over six years	Neighborhood park access: Cognitive function( +)
39	Besser,2021a [75]	Compared to individuals with no walking destinations in the 1-mile surrounding their residence, those with 716 walking destinations were 1.24 times more likely to have maintain/improved DSC score (Odds ratio: 1.24; 95% confidence interval: 1.03–1.45)	Processing speed: Neighborhood walking destinations were associated with( +), Social destination density(0), Proportion of land dedicated to retail(0), Street connectivity(0)
40	Finlay,2021 [20]	A significant positive associations between kernel density of senior centers, civic/social organizations, and cognitive function	Cognitive function: The kernel density of senior centers( +), Civic/Social organizations( +)
41	Finlay,2021a [76]	Results indicated that residing in neighborhoods with greater availability of local parks, access to recreational amenities, and business density was associated with higher levels of cognitive function	Cognitive function: The availability of local parks( +), Access to recreational amenities( +), Business density( +)
42	Hsu, 2021 [41]	The better cognitive function was related to higher city population density, higher safety in the community, more barrier-free sidewalks	Cognitive function: City population density( +), Safety in the community( +), Barrier-free sidewalks( +)
43	Jin, 2021 [77]	1.Among those with low genetic AD risk, the risk of cognitive impairment was lower in those living around higher greenness (contemporaneous NDVI OR: 0.55, 95% CI: [0.34, 0.86]; Pinteraction: 0.071; annual average NDVI OR: 0.49, 95% CI: [0.31, 0.79]; Pinteraction: 0.040) 2.No significant association between green and cognitive impairment was observed in people with a high risk of inherited AD	Cognitive function: greenness:(+ ,for low genetic AD risk; 0,for high genetic AD risk)
44	Motohiro, 2021 [78]	The second quartiles of residential density showed significantly lower likelihoods of cognitive decline compared with the first quartiles (adjusted OR 0.36, 95% CI (0.19, 0.71))	Cognitive function: Residential density(-),Distance to community center(0)
45	Tani, 2021 [79]	In urban areas, compared with the lowest quartile of sidewalk coverage, the hazard ratio was 0.42 (95% CI, 0.54) for the highest quartile	Sidewalk installation: Incidence of dementia(-)
46	Thierry, 2021 [59]	1.Among White adults, worse neighborhood characteristics were associated with lower cognitive functioning 2.Among Black adults, poor perceived quality of one’s neighborhood was associated with worse cognitive functioning. Among Mexicans, perceived neighborhood uncleanliness was associated with lower cognitive functioning among those with less education, but higher cognitive functioning for those with higher levels of education	1.Neighborhood characteristics: Cognitive function(+ ,for White adults) 2.Perceived quality of one’s neighborhood: Cognitive function(+ ,for Black adults; + , for Mexicans with less education; -,for Mexicans with higher levels of education)
47	Bai, 2022 [60]	1.The relationship between main road network density and cognitive function was significant and negative (β < 0; *p* < 0.05); 2.Secondary road network density (β > 0; *p* < 0.05) and other road network density (β > 0; *p* < 0.05) had a positive correlation with cognitive function	Cognitive function: The main road network density(-), The secondary road network density and other road network density( +)
48	Cerin, 2022 [61]	Findings suggest that dense, interconnected urban environments with access to parks, blue spaces and low levels of air pollution may benefit cognitive health through cardiometabolic risk factors and other mechanisms not captured in this study	Cognitive health: Population density( +), Interconnected urban environments with access to parks( +), Blue spaces( +)
49	Finlay, 2022 [80]	1.The unequal distribution of amenities and hazards across neighborhoods may help account for considerable inequities observed in cognitive health among older adults 2.Neighborhood highway density was negatively associated with participants’ cognitive function	Cognitive function: Amenities( +), Neighborhood highway density (-)
50	H. 2022	1.The male participants with no access to recreation spaces had lower MMSE scores (β = -1.107, 95% CI: -1.531, -0.684, *p* < 0.001) 2.The female participants who lived far from a supermarket had significantly lower MMSE scores (Q3:β = -0.750, 95% CI: -1.266, -0.233, p adjusted = 0.036; Q4: β = -1.184, 95% CI: -1.745, -0.624, p adjusted < 0.001) than those who lived near a supermarket	1.Recreation spaces: Cognitive function(+ , for male participants); 2.The distance of supermarket: Cognitive function(-, for female participants)
51	Slawsky, 2022 [81]	Compared to low residential greenspace, high residential greenspace was associated with a reduced risk of dementia (HR = 0.76 95% CI: 0.59,0.98) in models adjusted for multiple covariates	Cognitive function: Residential greenspace ( +)
52	Sylvers, 2022 [63]	Living in neighborhoods with greater street connectivity (β = 0.15, *p* < 0.05), and positive perceptions of neighborhood traffic (*p* < 0.01) and parks (*p* < 0.05), were associated with higher cognitive function	Cognitive function: Street connectivity( +), Positive perceptions of neighborhood traffic and parks( +)
53	Chan 2023 [37]	Residents aged 80 and younger in neighborhoods with a higher land-use mix and more public transport terminals exhibited better cognition. Only the number of community centers in a neighborhood was positively associated with cognition for people older than 80	Cognitive function: Land-use mix( +), Public transport terminals( +), community centers( +)
54	Godina, 2023 [82]	1.We observed no significant association between overall percent greenspace and risk of mild cognitive impairment or dementia and mostly null results for forest and greenspace diversity 2.Forest greenspace was associated with lower odds of MCI (OR quartile 4 versus 1: 0.54, 95% CI: 0.29 –0.98) and greenspace diversity was associated with lower hazard of incident dementia (HR tertile 2 versus 1: 0.70, 95% CI = 0.50–0.99)	1.Overall greenspace: MCI(0), Dementia(0); 2.Forest greenspace: MCI(-) 3.greenspace diversity: Dementia(-)
55	Ho, 2023 [38]	For circular buffers, higher percentage of building coverage, higher land use mix and more community/transportation/leisure facilities were negatively associated with dementia	Dementia: Building coverage(-), Land use mix(-), Community/Transportation/Leisure facilities(-)
56	Kim, 2023 [64]	Perceived walkability was associated with a 0.04 point higher cognitive function score through walking (*p* = 0.006)	Cognitive function: Perceived walkability( +)

*AD* Alzheimer’s disease, *CI* Confidence interval, *MoCA* Montreal Cognitive Assessment, *GIS* Geographic Information System, *MMSE* Mini-Mental State Examination, *LMIC* low- and middle-income countries, *CASI* Cognitive Abilities Screening Instrument, *DSC* Digit Symbol Coding, *TMT* Trail Making Test, *HVLT-R* Hopkins Verbal Learning Test-Revised, *RBANS* Repeatable Battery for the Assessment of Neurocognitive Status, *APOE* apolipoprotein E, *SDD* Social and walking destination density, *WDD* Walking destination density (SDD, WDD), *CFAS II* Cognitive Function and Ageing Study II, *MCI* Mild cognitive impairment, *HR* Hazard Ratio, *SD* Standard Deviation

#### Association between residential/population density and cognitive function

Based on previous studies, population density may have a positive effect on cognitive function in older adults, but residential density does not appear to affect cognitive function in older adults. A total of five studies investigated the effects of residential/ population density on cognitive function in older adults. Specifically, Hsu et al. found that better cognitive function in older adults was associated with higher city population density [41]. Cerin et al. showed that higher population density is beneficial to the cognitive health of older adults [61]. In addition, a study from Japan showed that higher hilly environments and residential density may predict a decline in cognitive function in rural older adults [78]. However, the study by Besser et al. did not find a significant effect of the proportion of residential land use on overall cognition and processing speed in older adults [44]. Neither subjective nor objective measures of residential density by Ng et al. found significant effects on global cognition, immediate and delayed memory recall, visuospatial/construction, and language skills in older adults [42].

#### Association between neighborhood built environment design and cognitive function

Most of the studies reviewed showed a significant positive correlation between cognitive function and street connectivity and walkability in older adults, but a possible negative correlation with neighborhood integration. For example, Ng et al. showed that objectively measured street connectivity had significant positive correlations with global cognition and immediate and delayed memory in older adults, but subjective street connectivity had no significant correlations with global cognition, immediate and delayed memory, attention, language, or visuospatial/constructional [42]. This suggests that different measures may influence the relationship between street connectivity and older adults' cognition. Neighborhood integration measures how many turns or choice points a person must go through to access all of the locations in a finite system. A study from the United States showed that for older adults without mild AD, higher levels of neighborhood integration predicted greater declines in attention and verbal memory during a two-year follow-up period, yet higher neighborhood connectivity predicted less decline in attention [32].

A Japanese study found a significant negative correlation between street integration and the odds of having a cognitive impairment [48]. However, Clarke et al. showed that the presence of crosswalks around homes and the presence of discontinuous sidewalks did not significantly affect the decline in cognitive function in older adults over time [31]. The results of Tani et al. showed a significant negative association between sidewalk coverage and the risk of dementia in urban areas [49]. In addition, Kim et al. showed a significant positive correlation between perceived walkability and cognitive function [64].

#### Association between public transportation distance and cognitive function

The majority of previous studies have shown that the distance of the residence from a major road is negatively associated with cognitive function in older adults, while the number of public transportation stops around the residence is positively associated with cognitive function. Specifically, a total of four studies investigated the effect of distance from a major road on cognitive function in older adults, with three studies showing a negative association with distance from a major road and one study showing no effect. For example, Wellenius et al. showed that living near a major roadway harms cognition in older adults [29]. A study from Canada showed that the proximity of residences to roads was associated with a higher incidence of dementia [34]. However, it was also shown that the distance between the residence and the bus stop does not affect the cognitive function of Chinese older adults [68].

In addition, two studies investigated the effect of the number of public transportation stops around the residence on the cognitive function of older adults, and both studies showed that the number of public transportation stops had a positive effect on cognition. For example, Clarke et al. found that living in neighborhoods with community centers and public transportation stops resulted in a slower rate of cognitive decline over time in older adults [31]. Chan et al. showed that in communities with more public transportation terminals, residents aged 80 and younger would show better cognitive performance [37].

#### Association between destination accessibility and cognitive function

The effects of destination accessibility on cognitive function and dementia in older adults had mixed results. Among them, Guo et al. showed that library accessibility was positively associated with cognitive function in older adults and negatively associated with dementia [36]. A study by Tani et al. reported a significant negative association between the availability of healthy food stores around the home and the incidence of dementia [49]. In addition, a previous study showed that female participants who lived away from the supermarket had significantly poorer cognitive function than those who lived near the supermarket [62].

However, some studies have also shown no significant association between destination accessibility and cognitive function and dementia in older adults. A study of older adults in Singapore showed that subjective and objective assessments of destination accessibility had no significant effects on overall cognition, immediate and delayed memory, attention, language, or visuospatial/constructional in older adults [42]. In addition, studies of older adults in China have shown that the availability of healthcare services has no significant effect on either cognitive function or decline in cognitive function in older adults [68]. The relationship between accessibility to destinations around residences and cognitive function in older adults needs to be further explored in the future.

#### Association between neighborhood built environment diversity and cognitive function

Such as land use mix, and neighborhood resource indicators that may reflect the diversity of a neighborhood's built environment. Most studies concluded that a higher land use mix and rich neighborhood resources in residential communities may have a positive impact on the cognitive functioning of older adults. In terms of land use mix, a cross-sectional study revealed that subjective measures of land use mix diversity were significantly and positively associated with global cognition, immediate and delayed memory recall, visuospatial/constructive ability, and language [42]. A cross-sectional study from Hong Kong, China, also showed that older adults living in communities with a higher land use mix exhibited better cognitive performance [37]. Moreover, A previous study showed that living in an area with a high land use mix was significantly associated with nearly 60% lower odds of dementia, but there was no linear trend in cognitive impairment [33]. However, studies have also shown that living in areas with a high mix of land uses is associated with nearly 30% lower odds of cognitive impairment [35].

Neighborhood resources include some basic infrastructure and services for daily living, such as community centers, supermarkets, health care services, recreational facilities, libraries, grocery stores, and fast food restaurants. A longitudinal study from the United States showed that living in a community with more accessible recreational facilities and commercial density was associated with higher levels of cognitive functioning in older adults [20]. In addition, there was a significant positive correlation between the kernel density of senior centers, civic/social organization, and cognitive function [55]. Liu et al. showed that the availability of community centers was significantly associated with an 8% reduction in the probability of dementia [40]. However, some studies have also concluded that the relationship between the availability of neighborhood resources and cognitive function in older adults is not significant [30, 68].

#### Association between blue/green spaces and cognitive function

In general, green space has a positive effect on cognitive function in older adults, but fewer studies have addressed the relationship between blue space and cognition, and the results have been mixed, and further exploration is needed in the future. More than half of the included studies described the relationship between green/blue space and cognition in older adults, but only three of these studies described the effect of blue space on cognition [55, 61, 62].

First of all, in terms of green space, the results of Aitken et al. showed that the highest neighborhood greenness was associated with 20% lower odds of AD compared to the lowest neighborhood greenness [57]. The study by Besser et al. reported a positive effect of higher park accessibility on cognitive function [74]. A study by Slawsky et al. found that residences with a higher proportion of green space were associated with a reduced risk of dementia compared to a low proportion of green space [81]. Additionally, Godina et al. showed that forested green space was associated with lower odds of mild cognitive impairment (MCI) and that green space diversity was associated with a lower risk of dementia events, but no significant association was observed between overall green space percentage and risk of MCI or dementia [82]. A longitudinal study showed that among people with a low risk of genetic AD, those who lived in a more green surrounding had a lower risk of cognitive impairment. However, no significant association between greenness and cognitive impairment was observed in people with a high risk of genetic AD [77].

Second, in terms of blue space, a cross-sectional study from Australia showed that living in an environment with blue spaces is beneficial to the cognitive health of older adults [61]. However, a study from the UK showed that the availability of blue space was not associated with dementia [55]. Some scholarly research has shown that the presence of local blue space is associated with cognitive function but not MCI and that participants with any blue space within an 800-m buffer zone around their residence may have higher levels of cognitive performance than those without blue space nearby, but this association was only present in female participants [62].

#### Association between neighborhood built environment quality and cognitive function

Research on the relationship between neighborhood built environment quality and older adults' cognition remains limited, but most of the existing studies suggest that better neighborhood environmental quality may have a positive impact on cognition. The quality of the neighborhood built environment referred to in this study includes variables such as aesthetics, neighborhood physical disorder index, street disrepair index, neighborhood streetscape, traffic safety, and crime safety, Cleanliness. Thierry et al. showed that among white adults, poorer neighborhood characteristics were associated with lower cognitive function; among black adults, poor neighborhood quality was associated with poor cognitive function; and among Mexicans, perceived neighborhood uncleanliness was associated with lower cognitive function among those with less education but higher cognitive function among those with more education [59].

In terms of neighborhood physical disorder, higher neighborhood physical disorder was associated with poorer initial episodic memory and verbal fluency [72]. Similarly, Estrella et al. showed that female participants' perceived neighborhood physical disorder was associated with their poorer global cognitive and memory abilities [52]. A cross-sectional study showed that older adults' perception of greater neighborhood physical disorder was significantly associated with poorer episodic memory [50]. However, some studies did not find a relationship between neighborhood disorder and cognitive function [30, 46]. In terms of safety, a study from Taiwan, and China, showed that community safety was positively associated with older adults' perceptions [41]. The study by Cherrie et al. concluded that lower road traffic accident density was associated with better cognitive performance [67]. In addition, higher self-reported neighborhood pleasantness was associated with lower cognitive vulnerability, particularly among older adults living in the most rural and urban areas, but neighborhood streetscape was not associated with older adults' cognition [43]. And a study by Lee et al. found that the street disrepair index was significantly and negatively associated with cognitive function [46].

## Discussion

The purpose of this study was to systematically review the relationship between the neighborhood built environment and cognitive function in older adults. A total of 56 studies met the inclusion criteria for this study, including 31 cross-sectional studies, 23 longitudinal studies, 1 cross-sectional study design combined with a case–control study, and 1 longitudinal study design combined with a case–control design. The study focused on seven aspects of neighborhood built environment indicators divided into residential/population density, neighborhood built environment design, public transportation distance, the density of public transportation stops, destination accessibility, neighborhood built environment diversity, blue/green space, and neighborhood built environment quality. Most of the studies reviewed showed that these built environment indicators showed varying degrees of correlation with cognitive function of older adults.

In terms of residential/population density, the reviewed studies showed a significant positive association between population density and older adults' cognition [61], but residential density may not affect cognitive function [42]. At present, the specific mechanisms by which population density affects cognitive functions in older adults are unclear. Previous studies have shown that social interactions are associated with cognitive function in older adults [83, 84]. The size of the population density may cause changes in cognitive function by affecting the social interactions of older people, and it is suggested that the specific pathways through which population density affects cognitive function in older people should be analyzed from the perspective of social interactions in the future. In terms of neighborhood built environment design, the study found that older adults' cognitive function was significantly and positively related to street connectivity and walkability, but negatively related to street integration [32, 64]. Neighborhood street integration is a measure of the number of turns required to travel between two points. Higher street integration means fewer turns are required in practice. When street integration is higher, the cognitive engagement required for walking is lower, which is not conducive to cognitive training for older adults [32]. However, the higher the neighborhood street connectivity and walkability, the better it is for older adults to engage in walking activities. For example, higher intersection density provides more potential paths for walking and facilitates travel for older adults, and higher levels of physical activity positively affect [85].

Regarding the distance of homes from public transportation, the cognitive function of older adults was negatively and significantly correlated with the distance of homes from major roads, but positively and significantly correlated with the number of public transportation stops [31, 34]. The closer a community is to major roadways, the greater the risk of individual exposure to noise and traffic-related air pollution [86], which may affect the cognitive health of older adults. However, a higher number of public transportation stops around the community may increase opportunities for older adults to travel and improve their physical activity levels, which in turn promotes cognitive health [87]. Studies on the relationship between older adults' cognition and destination accessibility have yielded mixed results due to differences in region, sample size, assessment methods, and destination variables [49, 68], and future research is needed to standardize the criteria for destination accessibility assessment and further explore the impact of destination accessibility on older adults' cognitive function.

Regarding the diversity of the neighborhood built environment, older adults' cognition was positively correlated with the land use mix, and most studies showed a positive correlation with neighborhood resources as well [20, 37]. Higher land use mix and neighborhood resources can provide opportunities for older adults to engage in a variety of activities while providing an interactive environment for cognitively stimulating activities, which in turn promotes cognitive health [11, 33]. In terms of blue-green space, most studies analyzed the relationship between greenness around the neighborhood, park area, and distance from the park and cognition, and the results showed that green space was significantly and positively related to cognition, but the effect of blue space on older adults' cognition needs to be further explored [62, 81]. Previous studies have shown that exposure to green space can reduce individual stress, benefit physical and mental health, and potentially promote cognitive development by reducing air pollution [88, 89]. In addition, Detweiler et al. suggest that green space has a positive impact on the long-term care and rehabilitation of older adults and can be used to improve the quality of life of the global aging population [90]. Regarding the quality of the neighborhood built environment, research suggests that the better older adults' perceptions and objective measures of environmental quality, as well as a safe and clean neighborhood environment, the better older adults' cognitive function is likely to be [41, 50].

In a rapidly aging society, it is essential to provide older adults with a neighborhood built environment that is suitable for aging in place and that maintains cognitive health [91, 92]. Research has generally shown a significant correlation between neighborhood built environment and cognitive functioning in older adults, but future research on this topic needs to be more comprehensive in the future. First, the causal effect of the built environment on cognitive functions is not clear because previous studies have mainly utilized cross-sectional study designs. It is recommended that future studies adopt a longitudinal study design to further explore the relationship between the built environment and cognitive function in older adults. Second, some studies suggest that physical activity partially mediates the relationship between the built environment and cognitive function. For example, studies have shown that transportation and recreational physical activity have significant mediating effects between land use mix and cognitive function [42]. In addition, air quality may also mediate the relationship between the built environment and cognitive function [34]. Therefore, the moderating role of mediating variables (e.g., physical activity, and air quality) may be considered in future studies of the mechanisms and pathways of the built environment's effects on cognitive function in older adults. Third, the current standards for assessing environmental exposures are not uniform, and different standards for assessing the built environment may contribute to differences in results, suggesting the use of standardized measures in the future. Fourth, previous research has focused on analyzing the relationship between the objective built environment and cognition, but older adults' perceptions of the built environment are also important. The perception of the neighborhood built environment may be a more proximate determinant of individual health outcomes than objective measures, as it reflects the individual's life experiences [93]. Therefore, it is suggested that a combination of subjective and objective methods may be considered in the future to further analyze the impact of the neighborhood built environment on older adults' cognition. In addition, previous studies have analyzed the relationship between a single built environment element and cognitive function by using a single evaluation index of the built environment element, and the relationship between a comprehensive evaluation index of the built environment and cognitive function of older adults is missing. In future studies, comprehensive indicators of the built environment, such as the walkability index, can be introduced to further analyze the relationship between the built environment and cognitive function in older adults.

The strength of this study is that it provides a systematic and comprehensive review of the effects of the built environment on cognitive function in older adults and analyzes the relationship between different built environment indicators and cognition in older adults, which provides some reference basis for the development of intervention programs to improve cognitive function in older adults. However, there are some limitations in this study. First, the study only included published literature in English and not in other languages, which may have some selection bias. Second, we were unable to conduct a quantitative synthesis due to the large heterogeneity of built environment assessment criteria and study populations among the different studies. For example, there is a huge variability in the indicators used to assess environmental exposure. In the case of green space, some studies chose only the area of parks to represent the level of green space around the community [30], while some studies used the NDVI [45]. In addition, the spatial unit of analysis for assessing environmental exposure varied widely, for example, some studies assessed the built environment within a 500-m buffer zone around the community [66], while some studies used the 2000-m buffer zone [81]. Therefore, it is recommended that future research should develop criteria for assessing exposure to the built environment to provide a quantitative synthesis for future research on this topic. Third, not all included studies used objective assessments of the built environment and cognitive functioning, and some studies used self-reported questionnaires, so the questionnaire results may have produced some bias. Fourth, it has been suggested that different spatial units of analysis used in assessing individual exposure to various environmental characteristics may influence the results between the built environment and health [94]. The use of spatial units of analysis in different studies has not been analyzed in this study, which may have some impact on the results of this study.

## Conclusion

This systematic review provides a comprehensive overview of the effects of neighborhood built environment on cognitive function in older adults. In general, population density, street connectivity, walkability, number of public transportation stops around the residence, land use mix, neighborhood resources, green space, and quality of the neighborhood built environment were positively associated with cognitive functions of older adults, respectively. In contrast, street integration and the distance between the residence and the main road were negatively related to the cognitive function of the older adults. The results of this review may provide a scientific reference for relevant policymakers and for the development of intervention programs to improve cognitive function, and suggest that public health interventions may take into account neighborhood built environment aspects to promote cognitive health and reduce the risk of dementia in older adults.

### Supplementary Information

**Additional file 1: Table 1.** Study quality assessment.

## Data Availability

The dataset supporting the conclusions of this article is included within the article.
